# A prediction model of nodal metastasis in cN0 oral squamous cell carcinoma using metabolic and pathological variables

**DOI:** 10.1186/s40644-023-00552-z

**Published:** 2023-04-05

**Authors:** Feng Xu, Liling Peng, Junyi Feng, Xiaochun Zhu, Yifan Pan, Yuhua Hu, Xin Gao, Yubo Ma, Yue He

**Affiliations:** 1 Department of Nuclear Medicine, Shanghai Ninth People’s Hospital, Shanghai Jiao Tong University School of Medicine, Shanghai, China; 2 Shanghai Universal Medical Imaging Diagnostic Center, Shanghai, China; 3 Department of Oral Pathology, Shanghai Ninth People’s Hospital, Shanghai Jiao Tong University School of Medicine, Shanghai, China; 4 Department of Oral Maxillofacial & Head and Neck Oncology, Shanghai Ninth People’s Hospital, Shanghai Jiao Tong University School of Medicine, Shanghai, China

**Keywords:** ^18^F-FDG PET/CT, Oral squamous cell cancer, Nodal metastasis, Nomogram

## Abstract

**Background:**

The efficacy of ^18^F-fluorodeoxyglucose (^18^F-FDG) Positron Emission Tomography/Computed Tomography(PET/CT) in evaluating the neck status in clinically node-negative (cN0) oral squamous cell carcinoma(OSCC) patients was still unsatisfying. We tried to develop a prediction model for nodal metastasis in cN0 OSCC patients by using metabolic and pathological variables.

**Methods:**

Consecutive cN0 OSCC patients with preoperative ^18^F-FDG PET/CT, subsequent surgical resection of primary tumor and neck dissection were included. Ninety-five patients who underwent PET/CT scanning in Shanghai ninth people’s hospital were identified as training cohort, and another 46 patients who imaged in Shanghai Universal Medical Imaging Diagnostic Center were selected as validation cohort. Nodal-status-related variables in the training cohort were selected by multivariable regression after using the least absolute shrinkage and selection operator (LASSO). A nomogram was constructed with significant variables for the risk prediction of nodal metastasis. Finally, nomogram performance was determined by its discrimination, calibration, and clinical usefulness.

**Results:**

Nodal maximum standardized uptake value(nodal SUVmax) and pathological T stage were selected as significant variables. A prediction model incorporating the two variables was used to plot a nomogram. The area under the curve was 0.871(Standard Error [SE], 0.035; 95% Confidence Interval [CI], 0.787–0.931) in the training cohort, and 0.809(SE, 0.069; 95% CI, 0.666–0.910) in the validation cohort, with good calibration demonstrated.

**Conclusions:**

A prediction model incorporates metabolic and pathological variables has good performance for predicting nodal metastasis in cN0 OSCC patients. However, further studies with large populations are needed to verify our findings.

**Supplementary Information:**

The online version contains supplementary material available at 10.1186/s40644-023-00552-z.

## Background

Accurate N staging is critical for oral squamous cell carcinoma(OSCC) patients with clinically node-negative (cN0) neck. When patients have unilateral nodal metastasis, the overall survival may be reduced by 50%. If bilateral cervical nodal metastasis occurs, the survival time can be further halved [1]. Neck dissection(ND) thus maximizes survival probability but leads to over-treatment, which is accompanied by poorer health-related quality of life in approximately 75% of patients [2]. Recently, a prospective diagnostic study of ^18^F-fluorodeoxyglucose (^18^F-FDG) Positron Emission Tomography/Computed Tomography(PET/CT) in OSCC patients with cN0 or cN + neck showed that ^18^F-FDG PET/CT achieved a sensitivity and specificity of 83.3% and 84.8% in detecting cervical lymph node metastases [3], meanwhile, most of the OSCC patients (73.3%) in their study had no lymph node metastasis. It is no doubt that early OSCC patients without nodal metastasis can benefit from the strategy of “wait and watch surveillance” after resection of the primary tumor without ND [4]. Therefore, it is important for us to find an efficient way to predict the occult nodal metastasis in cN0 neck accurately. Although studies indicated that ^18^F-FDG PET [5] or PET/CT [6] could detect occult neck metastasis better than CT/MR imaging in cN0 OSCC patients, it was reported that its utility for detecting cervical nodal metastasis is limited in early OSCC [7]. A meta-analysis conducted by Kim et al. [8] showed the low sensitivity and moderate specificity of ^18^F-FDG PET/CT for the detection of cervical lymph node(LN) metastasis in cN0 HNSCC(head and neck SCC) patients. Therefore, ^18^F-FDG PET/CT is currently not sufficiently reliable to avoid elective treatment of the neck.

Previous reports indicated that pathological variables of primary tumor(OSCC) such as depth of invasion(DOI) [9, 10], pathological tumor size [11], perineural invasion(PNI) [12] were also widely used for prediction of occult nodal metastasis in cN0 OSCC patients. However, some of them still got unsatisfying results [13].

To the best of our knowledge, no studies tried to develop a prediction model for nodal metastasis in cN0 OSCC patients by using metabolic and pathological variables. The goal of this study was to determine whether the combination of metabolic and pathological variables could further improve the prediction of nodal metastasis in cN0 OSCC patients.

## Materials and methods

### Patients

**Fig. 1:**
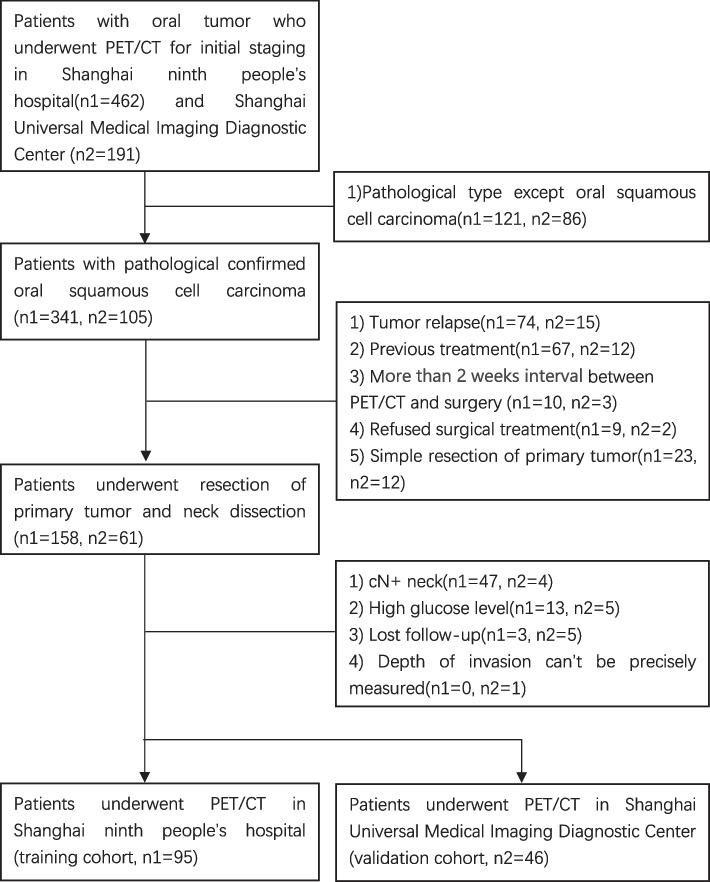
The workflow of patient selection

We retrieved data on all consecutive patients with oral tumor who underwent preoperative ^18^F-FDG PET/CT in Shanghai ninth people’s hospital and in Shanghai Universal Medical Imaging Diagnostic Center (from January 2018 to May 2022). The inclusion criteria were defined as follows: 1) ^18^F-FDG PET/CT was performed within two weeks before surgery. 2)Surgical resection of primary tumor and neck dissection. 3)The primary tumor was pathologically confirmed OSCC, and staged according to the latest(8^th^) Edition of the American Joint Committee on Cancer/Union for International Cancer Control(AJCC/UICC) TNM staging system [14], 4)Patients with cN0 neck, 5)Patients were followed up for at least 6 months after operation. Patients who had tumor relapse, previous treatment, or high blood glucose level (> 200 mg/dL) during the PET/CT scan were all excluded. According to the criteria, ninety-five patients imaged in Shanghai ninth people’s hospital were identified as the training cohort and another 46 patients scanned in Shanghai Universal Medical Imaging Diagnostic Center were selected as the validation cohort. All patients received surgical treatment and postoperative follow-up in Shanghai ninth people’s hospital. Details of patient selection were shown in Fig. 1.

In the training cohort, 52 patients with malignancies of tongue, followed by gingival (nineteen patients), buccal mucosa (ten patients), floor of mouth (nine patients) and palate cancers(five patients). ND level on the affected side were as follows: I-II:I-III:I-IV:I-V = 7:33:9:46, and ten patients underwent contralateral ND(I:I-II:I-III:I-IV:I-V = 1:2:4:2:1). Nodal metastases were histologically confirmed in 38 patients (40.0%), eight of them were found to have bilateral nodal metastasis. In the validation cohort, sixteen patients with malignancies of buccal mucosa, followed by tongue (fourteen patients), gingival (nine patients) and floor of mouth (seven patients). ND level on the affected side were as follows: I-II:I-III:I-IV:I-V = 1:21:3:21, and eight patients underwent contralateral ND(I-II:I-III = 2:6). Nodal metastases were histologically confirmed in 14 patients (30.4%), one of them was found to have bilateral nodal metastasis. No distant metastasis was found in the 141 patients by preoperative evaluation.

### Imaging technique

All patients were fasted at least 6 h before being injected with ^18^F-FDG. PET/CT scanning was performed on either Ingenuity TF (Philips Medical systems, Cleveland, USA) in Shanghai ninth people’s hospital or Biograph mCT (Siemens Healthcare, Erlangen, Germany) in Shanghai Universal Medical Imaging Diagnostic Center. PET/CT images were acquired from the base of the skull to the mid-thigh or foot approximately 60 min after the injection with ^18^F-FDG. The detailed acquisition parameters are given in Supplementary Material Table 1.

### Metabolic variables of primary tumor and lymph node

In the training cohort, the volume of interest (VOI) of the primary OSCC tumors was automatically delineated with a threshold of 40% of maximum standardized uptake value(SUVmax) [15] on the Intellispace Portal workstation (Philips, Best, the Netherlands). Then, the VOIs were carefully reviewed by a nuclear medicine radiologist(F.X) with 15 years of experience, and the borders of the volume of interest(VOI) were adjusted manually to exclude adjacent physiological ^18^F-FDG-avid structures on PET images. SUVmax, SUVmean, metabolic tumor volume (MTV) and total lesion glycolysis (TLG = SUVmean × MTV) of primary tumors were calculated. The same radiologist visually reviewed PET/CT images of the ipsilateral and contralateral neck, the LN with the most intense uptake of ^18^F-FDG was carefully chosen (in case of no LN with higher SUVmax than background, the LN with the longest diameter on the affected side was chosen), and the nodal SUVmax were measured visually by placing regions of interest over the neck LNs. The short and long axis diameter of the LN was measured as well. Another nuclear medicine radiologist(L.P) with 10 years of experience performed the measurements on the syngo VB20P workstation (Siemens Healthcare, Erlangen, Germany) for the validation cohort in the same way.

### Pathological variables of the primary tumor

All 141 patients had a detailed pathological diagnosis of the primary tumor in their hospital records. One oral pathologist(Y.H) checked the pathological findings of the primary tumor, including grade of tumor, surgical margin, PNI, pathological tumor size, depth of invasion, and lymphovascular invasion. Finally, according to the 8^th^ edition of AJCC/UICC TNM staging system, the pathological T stage was determined by pathological tumor size and depth of invasion.

### Standard reference for nodal metastasis

The histopathological diagnosis (immune-histochemical staining with cytokeratin and traditional hematoxylin and eosin staining) was the standard of reference for the ND side. Follow-up was taken as reference for the contralateral side without ND. Postoperative complications, primary site status and cervical nodal status were followed up every month for at least 6 months after operation.

### Construction of prediction model

In the training cohort, least absolute shrinkage and selection operator(LASSO) logistic regression algorithm, with penalty parameter tuning conducted by five-fold cross-validation, was applied to select nodal-status-related variables with nonzero coefficients. Multivariable forward stepwise logistic regression modeling was then performed to identify significant variables associated with nodal metastasis. A prediction model of nodal metastasis, with combination of significant variables, was established by using logistic regression modeling. To provide a more understandable outcome measure, a nomogram was then constructed by using the significant variables derived from the training cohort.

### Validation of the model and clinical utility

Calibration curves were plotted via bootstrapping with 1000 resamples to assess the prediction model, accompanied by the Hosmer–Lemeshow goodness of fit test, a *P*-value of the Hosmer–Lemeshow test > 0.05 indicated a good fit. Predictive accuracy of the prediction model was validated using receiver-operating-characteristic (ROC) and quantified by the area under the curve (AUC) in the training cohort and validation cohort. Finally, Clinical utility of the nomogram was assessed by decision curve analysis.

### Statistical analyses

Statistical analyses were carried out using SPSS(version 17.0, SPSS Inc., Chicago, Illinois, USA), MedCal(version 19.6.1, MedCalc Software, Ostend, Belgium) and R software(version 4.2.0, http://www.r-project.org, R Foundation for Statistical Computing, Vienna, Austria). Data are presented as mean ± SD or median (followed by range) when the distribution of data was skewed. Qualitative parameters were analyzed using the Fisher’s exact test. The LASSO logistic regression algorithm was carried out by using the “glmnet” package. Nomogram and calibration curves were performed with the “rms” package. Decision curve analysis was generated using the “rmda” package. A two-tailed test with *P* < 0.05 represented a statistically significant difference.

## Results

Twenty clinicopathological and metabolic variables in the training cohort and validation cohort were summarized in Table 1. In the training cohort, significant differences were found in clinical T stage, grade of tumor, pathological tumor size, depth of invasion, pathological T stage, PNI, tumor SUVmax, tumor SUVmean, tumor MTV, tumor TLG, nodal short diameter, and nodal SUVmax between patients with or without nodal metastasis.

**Table 1:** Patient Characteristics

	Training cohort(*n* = 95)			Validation cohort(*n* = 46)		
Variables	Nodal metastasis ( −) *n* = 57	Nodal metastasis ( +) *n* = 38	*P*	Nodal metastasis ( −) *n* = 32	Nodal metastasis ( +) *n* = 14	*P*
**Age**(year)	63.6 ± 12.4	60.6 ± 11.7	0.277	61.0 ± 12.3	65.0 ± 8.1	0.226
**Gender**(male:female)	42:15	33:5	0.123	22:10	11:3	0.496
**Drinker**(No vs Yes)	47:10	26:12	0.112	23:9	13:1	0.112
**Smoker**(No vs Yes)	39:18	24:14	0.595	20:12	11:3	0.285
**Clinical T stage**						
I: II: III	23:28:6	4:26:8	**0.006**	11:19:2	2:8:4	0.077
**Grade of tumor**						
Low: Moderate: Poor	6:51:0	0:32:6	**0.001**	5:26:1	0:13:1	0.259
**Pathological Tumor size**(cm)	2.5(1.0–6.0)	3.8(1.0–6.0)	**0.000**	3.0(1.0–5.0)	3.3(2.0–7.0)	0.140
**Depth of invasion**						
≦5 mm: > 5 mm,≦10 mm: > 10 mm	20:16:21	3:2:33	**0.000**	12:6:14	0:5:9	**0.027**
**Pathological T stage**						
I: II: III	14:22:21	1:4:33	**0.000**	10:11:11	0:4:10	**0.025**
**Margin**(negative vs positive)	56:1	32:2	0.286	32:0	13:1	0.304
**Perineural invasion**(No vs Yes)	46:11	19:15	**0.011**	28:4	8:6	**0.022**
**Lymphovascular invasion**(No vs Yes)	57:0	36:2	0.157	32:0	14:0	
**Tumor SUVmax**	12.3(3.0–32.4)	13.7(3.7–31.7)	**0.020**	8.9(2.1–49.7)	8.8(1.4–27.6)	0.830
**Tumor SUVmean**	5.9(1.8–16.8)	7.6(2.9–19.3)	**0.015**	5.4(1.4–32.6)	4.8(0.8–17.5)	0.933
**Heterogeneity index**^**a**^	1.7(1.1–2.6)	1.7(1.3–2.0)	0.312	1.6(1.5–1.8)	1.7(1.6–1.9)	0.403
**Tumor MTV**(cm^3^)	4.3(0.8–50.7)	9.0(2.0–70.3)	**0.000**	4.6(1.1–26.6)	6.3(2.1–93.4)	0.050
**Tumor TLG**	28.7(3.3–411.6)	75.2(10.3–1052.7)	**0.000**	25.6(3.3–156.1)	37.3(8.3–463.0)	0.166
**Nodal long diameter**(cm)	0.9(0.4–1.3)	1.0(0.5–1.2)	0.135	0.9(0.6–1.2)	1.0(0.7–1.2)	0.933
**Nodal short diameter**(cm)	0.6(0.3–1.1)	0.7(0.4–1.1)	**0.000**	0.7(0.3–1.1)	0.8(0.4–0.9)	0.193
**Nodal SUVmax**	2.2(0.5–5.2)	3.7(1.1–10.6)	**0.000**	1.9(0.9–4.5)	2.66(1.1–15.7)	0.079

^a^Heterogeneity index = Tumor SUVmax/Tumor SUVmean

### Variable selection for constructing models

**Fig. 2:**
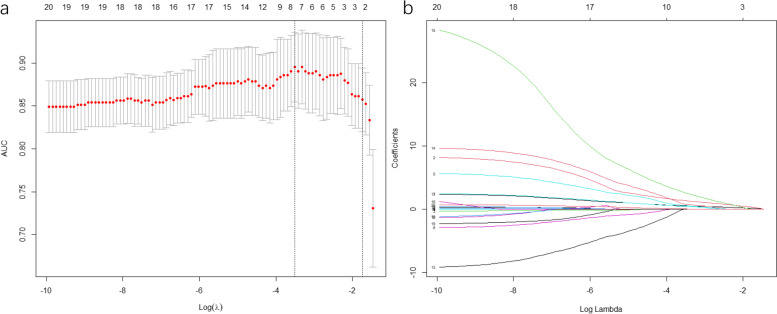
Variable selection by using the least absolute shrinkage and selection operator (LASSO) regression model. **a** Selection of tuning parameter (λ) in the LASSO model used fivefold cross-validation via minimum criteria. The left and right dotted vertical lines represent the optimal values of lambda when using the minimum criterion(λ value of 0.0299) and the one-fold standard error of minimum criterion(λ value of 0.1749), respectively. **b **LASSO coefficient profiles of the 20 selected variables

Using LASSO regression in the training cohort, 20 variables in the training cohort were reduced to 7 when using the minimum error criterion (Fig. 2). The selected variables including “nodal SUVmax”, “pathological T stage”, “drinker”, “grade of tumor”, “PNI”, “nodal short diameter” and “pathological tumor size”. In the multivariable forward stepwise logistic regression model, “drinker”, “grade of tumor”, “PNI”, “nodal short diameter” and “pathological tumor size” was justifiably removed.

### Construction, calibration and discrimination of prediction model

**Fig. 3:**
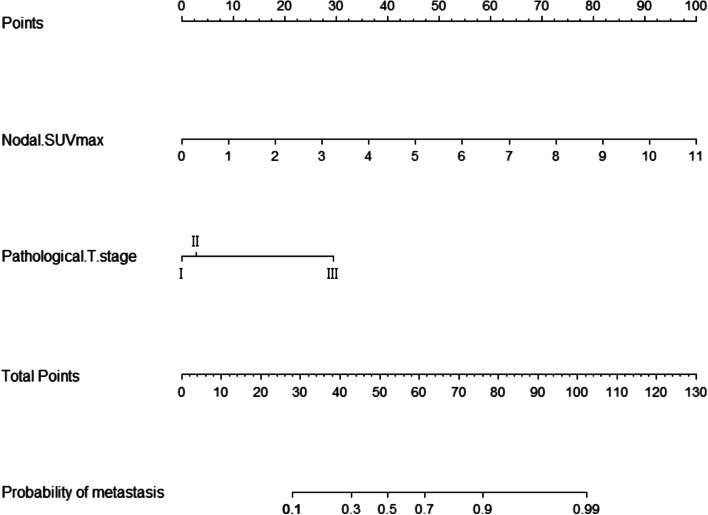
**a** The nomogram for predicting the risk of nodal metastasis in cN0 OSCC patients, based on nodal SUVmax and pathological T stage

“Nodal SUVmax” and “pathological T stage” were the significant variables for predicting nodal metastasis in the regression model (Table 2). The two significant variables were used to construct a nomogram(Prediction Model) for risk prediction of nodal metastasis (Fig. 3).

**Table 2:** Variables in logistic regression model

Variables	Odds Ratio (95% Confidence Interval)	*P*
Nodal SUVmax		0.001
≤ 2.71	Ref	
> 2.71	2.296(1.423–3.703)	
Pathological T stage		0.000
I	Ref	
II	1.299(0.114–14.863)	
III	14.746(1.707–127.396)

**Fig. 4:**
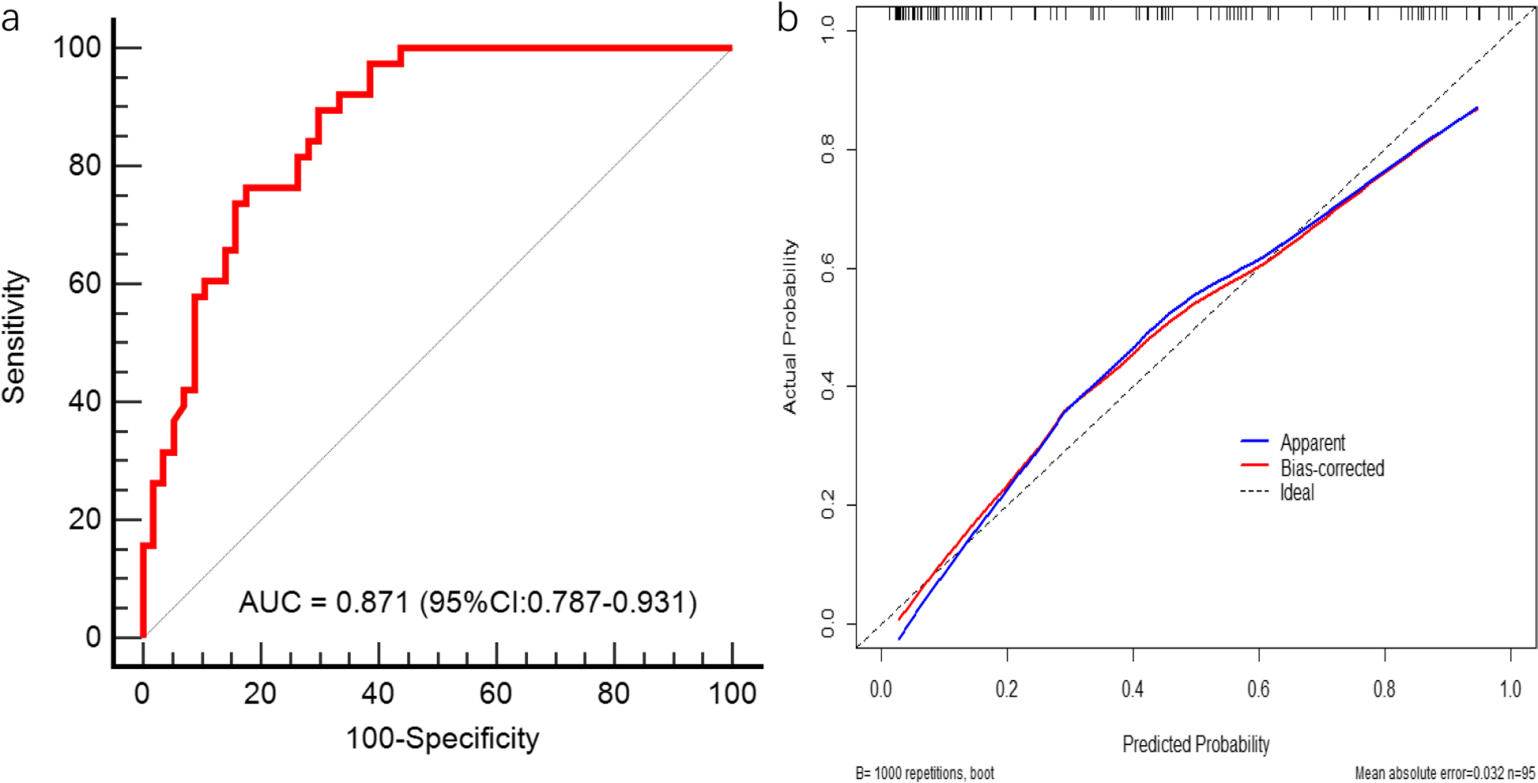
**a** Receiver operating characteristic curve of the nomogram(training cohort). **b** Calibration curve for the nomogram

In the training cohort, when nodal metastasis prediction performance was analyzed using ROC curve analysis, the AUC was 0.774(Standard Error [SE], 0.050; 95% Confidence Interval [CI], 0.667–0.859) for nodal SUVmax, 0.758 (SE, 0.042; 95% CI, 0.659–0.840) for pathological T stage, and 0.871 (SE, 0.035; 95% CI, 0.787–0.931) for prediction model (Fig. 4a). When choosing the prediction of 0.34 as optimal cutoff value, the prediction model achieved a sensitivity of 89.5%(34/38), a specificity of 70.2%(40/57), an accuracy of 77.9%(74/95), a positive predictive value of 66.7%(34/51), and a negative predictive value of 90.9%(40/44), calibration plot of the nomogram showed good agreement between predicted and observed nodal metastasis (Fig. 4b), the Hosmer–Lemeshow test yield a *P* value of 0.532, demonstrating a good fit.

**Fig. 5:**
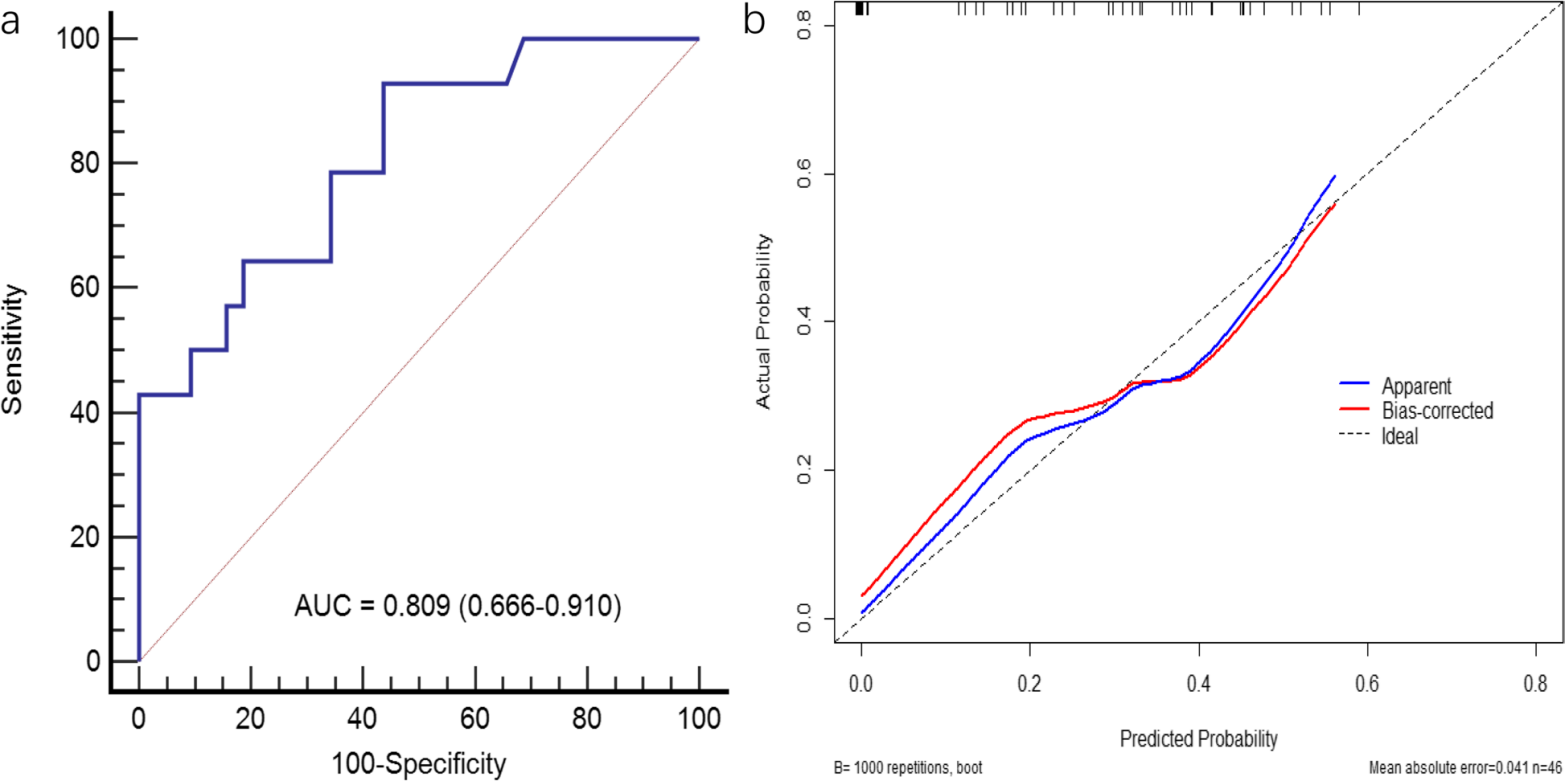
**a** Receiver operating characteristic curve of the validation cohort. **b** Calibration curve for the validation cohort

The good calibration was further confirmed in the validation cohort with AUC of 0.809(SE, 0.069; 95% CI, 0.666–0.910) (Fig. 5), the Hosmer–Lemeshow test yield a *P* value of 0.694.

### Clinical use

**Fig. 6:**
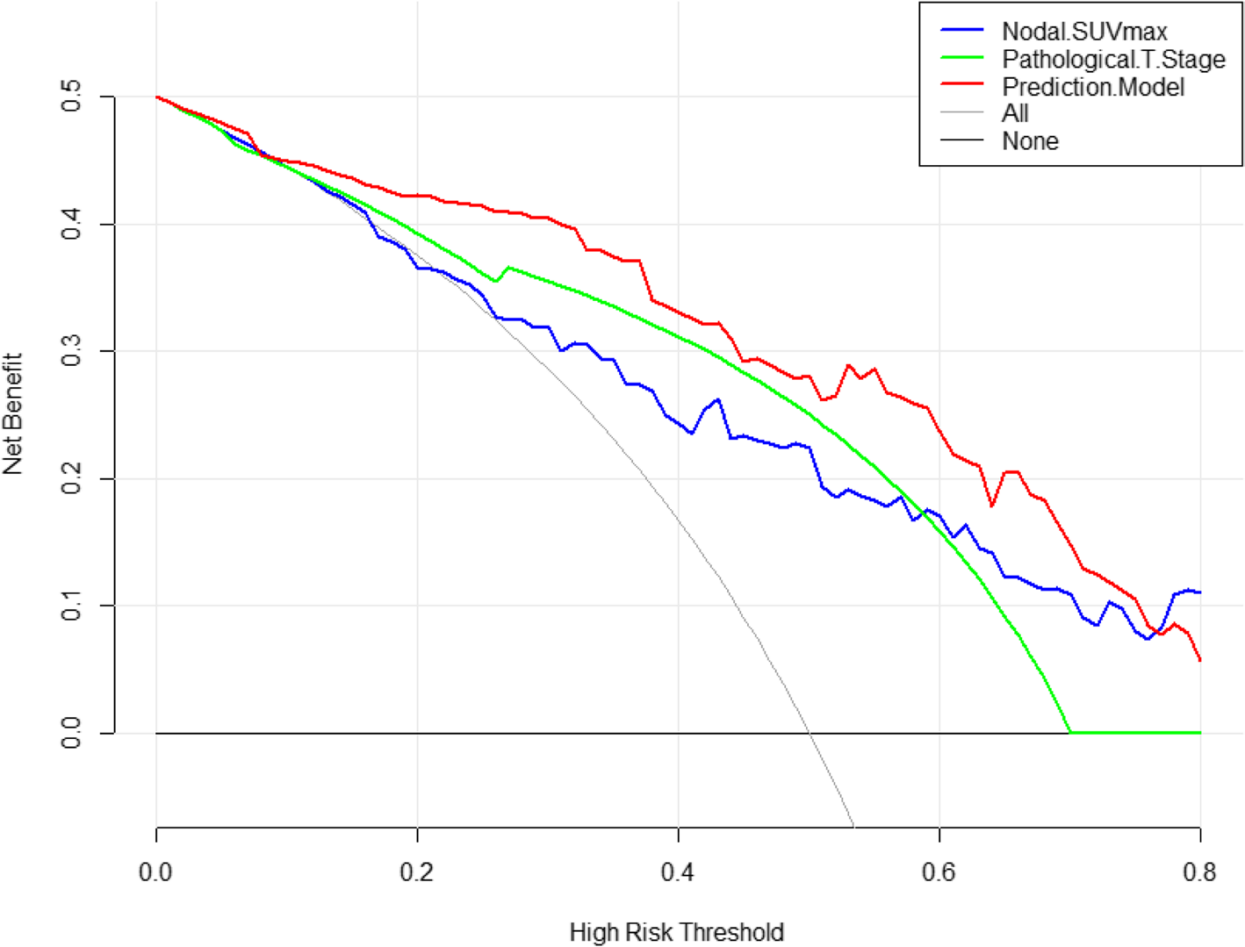
Decision curve showed that the prediction model presented with a better performance than the “nodal SUVmax”, “pathological T stage”, and “two extreme lines” (treat-none and treat-all) when the threshold probability was 0–0.8

**Fig. 7:**
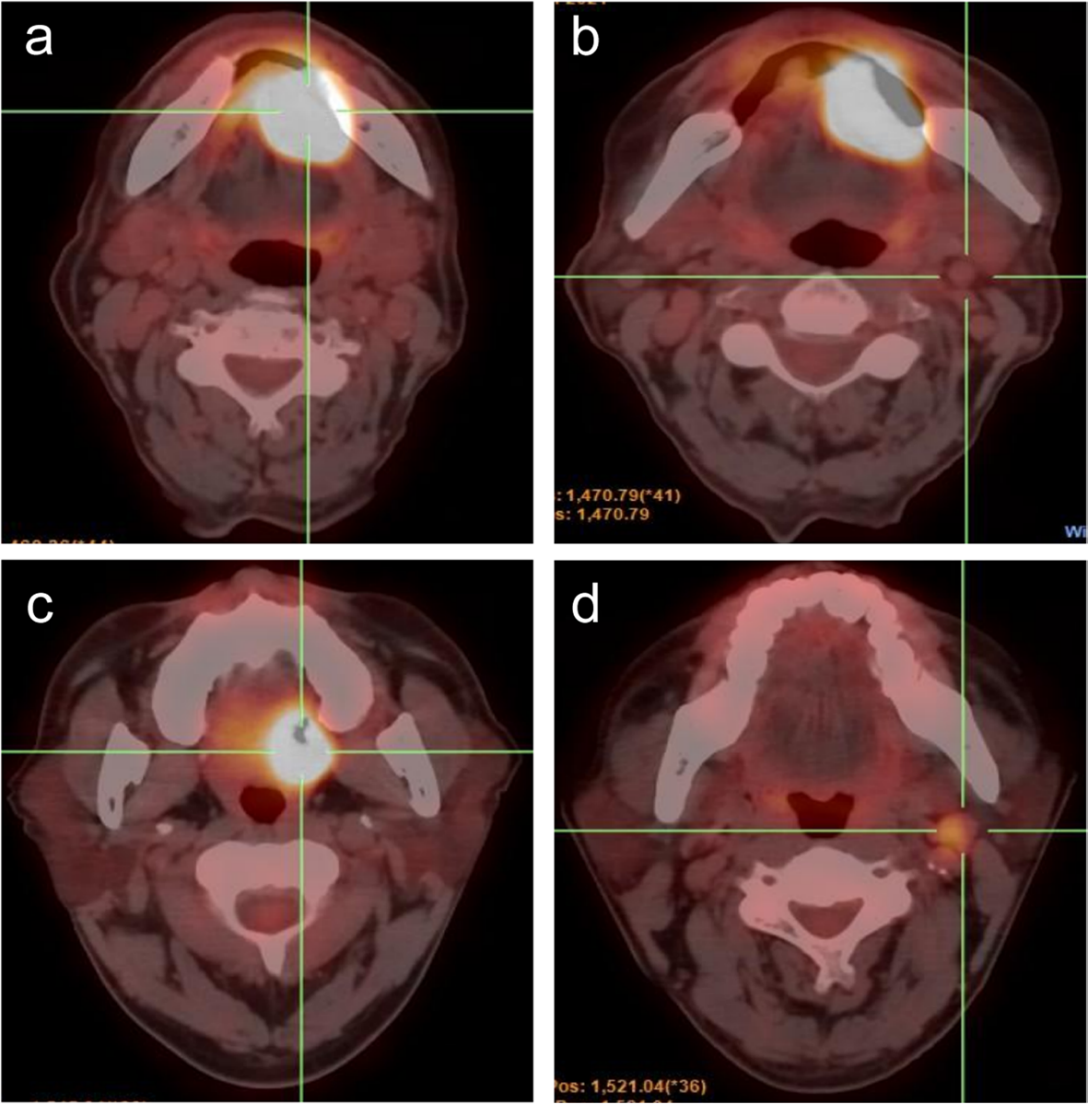
(a-b) A 63-year-old male with floor of mouth SCC. **a** PET/CT showed intense ^18^F-FDG uptake in the primary tumor(pathological T stage III, high probability of LN metastasis). **b** A LN with the most intense uptake of ^18^F-FDG was observed in the left neck side with SUVmax 1.76(Low probability of LN metastasis); The prediction model indicated the probability of nodal metastasis, which was confirmed by neck dissection (I-V). (c-d) A 55-year-old male with palate SCC. **c** PET/CT showed intense ^18^F-FDG uptake in the primary tumor(pathological T stage II, low probability of LN metastasis). **d** A LN with the most intense uptake of ^18^F-FDG was observed in the left neck side with SUVmax 4.62 (high probability of LN metastasis); The prediction model suggested probability of nodal metastasis, which was confirmed by neck dissection (I-V)

Decision curve showed that the prediction model presented with a better performance than the “nodal SUVmax”, “pathological T stage”, and “two extreme lines” (treat-none and treat-all) when the threshold probability was 0–0.8 (Fig. 6). Representative images for patients with cN0 neck correctly diagnosed by the prediction model were shown in Fig. 7.

## Discussion

In the present study, we tried to establish an efficient prediction model for nodal status based on 20 nodal status-related variables in cN0 OSCC patients. Finally, one metabolic variable(nodal SUVmax) and one pathological variable(pathological T stage) were selected to build the prediction model.

There were various reports on the association between metabolic variables of the primary tumor and nodal metastasis. Morand et al. [16]. found that higher SUVmax (≥ 9.5) of the primary tumor was associated with higher occurrence of metastatic nodal disease(AUC 0.651) in 71 cN0 or cN + OSCC patients. Bae et al. [17]. suggested that MTV(AUC 0.629) and TLG(AUC 0.626) of the primary tumor independently predicted nodal metastasis in 130 cN0 OSCC patients. However, some limitations in their studies should be mentioned. First, the nodal SUVmax, which represent the metabolism of LN, was not analyzed, second, the physiologic ^18^F-FDG uptake in the oral cavity, which may cause overestimation of tumor MTV and TLG, was not concerned, furthermore, their results were based on two different PET scanners. Lastly, most of all, the AUCs of the metabolic variables of the primary tumor were relatively low. In this study, patients in the training cohort (or validation cohort) underwent a same PET/CT scanner with same protocol, the borders of the VOI of the primary tumor were adjusted manually to exclude adjacent physiological ^18^F-FDG-avid structures on PET images, and nodal SUVmax were considered as well. Significant differences were observed in tumor SUmax, SUVmean, MTV, TLG and nodal SUVmax between cN0 OSCC patients with and without nodal metastasis in the training cohort (Table 1). LASSO regression indicated that nodal SUVmax was the only significant metabolic variable in predicting nodal metastasis, unfortunately, its AUC(0.774) was still dissatisfied. Yamazaki et al. [18] noted that detection of smaller volumes was limited by the capabilities of the PET/CT scanner resolution. PET/CT was found invalid if the lesion was smaller than 5 mm. Furthermore, some occult nodal metastases harbored a small amount of 18F-FDG uptake, while some inflammatory LNs or LNs close to the submandibular gland may got relative higher value of nodal SUVmax [19]. Therefore, nodal SUVmax should not be the sole criterion for predicting the presence of neck metastasis.

Pathological variables of primary tumor(OSCC) were also widely used to predict nodal metastasis in cN0 OSCC patients. As reported in the literature, the pathological tumor size [11], DOI [9, 10], and PNI [12] were important pathological variables which were associated with nodal metastasis in OSCC patients. In the latest(8^th^) Edition of the AJCC/UICC TNM staging system [14], DOI was introduced as a fundamental staging criterion to define T1, T2, and T3 categories as it correlates well with the risk of nodal metastasis and loco-regional recurrence, especially in tongue SCC [9, 10]. In the present study, pathological T stage was determined by pathological tumor size and DOI, as introduced by the 8^th^ Edition of the AJCC/UICC TNM staging system [14]. Interestingly, it was proved to be a significant predictor of nodal metastasis, with an AUC of 0.758, rather than pathological tumor size and DOI. We infer that pathological T stage, which combined pathological tumor size and DOI, maybe potentially more useful in predicting the nodal metastasis than the two variables separately. We believed it could serve as useful complementary predictive variables for nodal metastasis.

In the present study, a prediction model which was based on nodal SUVmax and pathological T stage was successfully built by using logistic regression modeling. It achieved a higher AUC(0.871) compared to nodal SUVmax and pathological T stage. Reports on the prediction model of nodal metastasis in cN0 OSCC patients were various, most of them were based on clinicopathological [20–24] and radiologic parameters [25]. Bur et al. [21] developed machine learning algorithms by using clinicopathologic data from 782 cT1-2N0 OSCC patients to predict pathologic nodal metastasis, they found the best classification performance was achieved with a decision forest algorithm (AUC = 0.840). Wang et al. [22]. constructed a prediction model for nodal metastasis based on molecular panel and clinicopathological factors in 112 cN0 OSCC patients, the model with the combination of CDKN2A, PLAU, T stage and pathological grade was the best in predicting lymph node metastasis (AUC = 0.807). Yuan et al. [25]. developed several machine learning models to predict occult nodal metastasis in 116 early-stage oral tongue SCC patients from preoperative MRI texture features, they found naïve Bayes model gave the best overall performance with an AUC of 0.802. Farrokhian et al. [24]. built a predictive model with XGBoost architecture in 634 early stage OSCC patients, they achieved an AUC of 0.840. Unfortunately, these methods described above were relatively complicated and they were not routinely used in clinical practice. Recently, Kallarakkal et al. [23]. established a predictive model based on the clinicopathological parameters only, including gender, tumor size, site, pattern of invasion and DOI, whereas, the AUC(0.752) they achieved was relatively low. We believe the combination of metabolic and pathological variables may be another useful way to predict the nodal metastasis in cN0 OSCC patients. The advantages of this model were as follows, first, the prediction model in the present study was trained and validated by data acquired from two different imaging centers, the subsequent calibration plot and ROC analysis illustrating a good discriminatory ability and predictive accuracy. Second, the nodal SUVmax and pathological T stage were easy to be obtained, which make the model simple to be established. Besides, preoperative PET/CT is also a good method for distant metastasis screening in OSCC patients. Finally, the model was useful to predict the probability of nodal metastasis for cN0 OSCC patients who underwent simple resection of the primary tumor without ND. Patients with negative results could rely on the high negative predictive value of the model (90.9%) to rule out nodal metastasis. However, caution should be exercised in patients with positive results as the positive predictive value (66.7%) was relatively low, leading to potential unnecessary neck dissections. Therefore, such patients should undergo more careful follow-up to confirm neck status.

Our study had several limitations. First, it is a retrospective study including selection bias. Second, the partial volume effect was not corrected for the LN, the value of nodal SUVmax may be underestimated. Third, the result of pathological T stage can only be obtained after surgery, which makes the pre-operative evaluation impossible. Lastly, the number of patients involved in the study was relatively small, further study with large number of patients is needed.

## Conclusion

We proposed a prediction model that incorporates the nodal SUVmax and pathological T stage to facilitate evaluation of LN status in OSCC patients with cN0 neck. To our knowledge, this is the first study which combined metabolic and pathological variables in predicting nodal metastasis in OSCC patients with cN0 neck. However, further studies with large populations are needed to verify our findings.

## Supplementary Information

**Additional file 1: SupplementaryMaterial Table 1.**

## Data Availability

Data available at supplementary file.

## Abbreviations

cN0: Clinically node-negative
OSCC: Oral squamous cell carcinoma
ND: Neck dissection
^18^F-FDG: ^18^F-fluorodeoxyglucose
PET/CT: Positron Emission Tomography/Computed Tomography
PNI: Perineural invasion
DOI: Depth of invasion
LASSO: Least absolute shrinkage and selection operator
SUVmax: Maximum standardized uptake value
AUC: Area under the curve
AJCC/UICC: American Joint Committee on Cancer/Union for International Cancer Control
OSEM: Ordered subsets expectation maximization
VOI: Volume of interest
MTV: Metabolic tumor volume
TLG: Total lesion glycolysis
