# Correction: A prediction model of nodal metastasis in cN0 oral squamous cell carcinoma using metabolic and pathological variables

**DOI:** 10.1186/s40644-023-00563-w

**Published:** 2023-05-19

**Authors:** Feng Xu, Liling Peng, Junyi Feng, Xiaochun Zhu, Yifan Pan, Yuhua Hu, Xin Gao, Yubo Ma, Yue He

**Affiliations:** 1 Department of Nuclear Medicine, Shanghai Ninth People’s Hospital, Shanghai Jiao Tong University School of Medicine, Shanghai, China; 2 Shanghai Universal Medical Imaging Diagnostic Center, Shanghai, China; 3 Department of Oral Pathology, Shanghai Ninth People’s Hospital, Shanghai Jiao Tong University School of Medicine, Shanghai, China; 4 Department of Oral Maxillofacial & Head and Neck Oncology, Shanghai Ninth People’s Hospital, Shanghai Jiao Tong University School of Medicine, Shanghai, China

**Correction: Cancer Imaging (2023) 23:34**

10.1186/s40644-023-00552-z

The original publication of this article contained an incorrect author contribution section. The incorrect and correct information is listed below. The original publication [1] has been updated.

**Incorrect**

NA designed the research, collected data, organized the data on the computer, did the analysis, interpretation, and identification, and wrote the draft manuscript. WT proposed the research concept, read the draft, reviewed, edited, supervised, and validated the final manuscript.

**Correct**

Study concept and design: FX, YM and YH. Acquisition of data: FX, LP, and YH. Analysis and interpretation of data XZ, JF, YP, and XG. Drafting of the manuscript: FX. Critical revision of the manuscript: YM, and YH.
