# Supplementary material for: A prediction model of nodal metastasis in cN0 oral squamous cell carcinoma using metabolic and pathological variables
Source: Cancer Imaging. 2023 Apr 5;23:34. doi: 10.1186/s40644-023-00552-z (PMC10074690; doi:10.1186/s40644-023-00552-z)
Supplement: Supplementary file 1 — Additional file 1: SupplementaryMaterial Table 1. [file 40644_2023_552_MOESM1_ESM.docx]

Supplementary Material Table 1

Image acquisition parameters.

| Acquisition  parameters | Ingenuity TF | | Biograph mCT | |
| --- | --- | --- | --- | --- |
|  | PET | CT | PET | CT |
| ^18^F-FDG activity  (MBq/kg) | **3.7** | **——** | **3.7-5.55** | **——** |
| Min/bed position | **1.5** | **——** | **1** | **——** |
| Crystal | **LYSO** | **——** | **LSO** | **——** |
| Matrix (pixels) | **169×169** | **1024×1024** | **200×200** | **512×512** |
| Slice thickness (mm) | **3** | **3** | **5** | **3** |
| Voxel size | **4🞨4🞨4 mm^3^** | **——** | **4.1🞨4.1🞨3.0 mm^3^** | **——** |
| Reconstruction | **OSEM**  **3 iterations and 33 subsets** | **——** | **OSEM**  **2 iterations and 21 subsets** | **——** |
| Slices | **——** | **128** | **——** | **64** |
| Voltage (kV) | **——** | **120** | **——** | **120** |
| Tube current (mA) | **——** | **150-200** | **——** | **100-180** |
